# Posttraumatic stress disorder and depression of Chinese medical staff after 2 years of COVID‐19: A multicenter study

**DOI:** 10.1002/brb3.2785

**Published:** 2022-10-19

**Authors:** Yifang Liu, Wenning Fu, Li Zou, Jing Wen, Pu Zhang, Jun Zhang, Xue Bai, Jing Wang, Jing Mao

**Affiliations:** ^1^ School of Nursing, Tongji Medical College Huazhong University of Science and Technology Wuhan China; ^2^ Reproductive Medical Center Renmin Hospital of Wuhan University & Hubei Clinic Research Center for Assisted Reproductive Technology and Embryonic Development Wuhan China; ^3^ Department of Neurology Zhongnan Hospital of Wuhan University Wuhan China; ^4^ Department of Cardiology, Taihe Hospital Wuhan University of Medicine Shiyan China; ^5^ Department of Endocrinology, Taihe Hospital Wuhan University of Medicine Shiyan China

**Keywords:** COVID‐19, depression, medical staff, posttraumatic stress disorder

## Abstract

**Background:**

In December 2019, coronavirus disease (COVID‐19) was first reported in Wuhan, China, and has had a negative psychological impact on the medical staff. However, the long‐term psychological effects of COVID‐19 were still unclear. We aimed to assess the posttraumatic stress disorder (PTSD) and depression among medical staff 2 years after COVID‐19 pandemic in Wuhan, China.

**Methods:**

We conducted a multicenter study in five general hospitals in Wuhan, China. PTSD was assessed using the PTSD Checklist‐5. Depression was measured by the Center for Epidemiologic Studies Depression Scale. Multivariate adjusted logistic regression models were used to evaluate the association among demographic variables, depressive indicators, and PTSD.

**Results:**

In a sample of 1795 medical staff, 295 (16.40%) participants reported PTSD and 329 (18.30%) reported depression. After multivariate adjusted logistic regression analyses, participants involved in COVID‐19 clinical work, unsafe working environment, poor doctor–patient relationship, unhealth status, work dissatisfaction, and low family support were at a high risk for PTSD and depression 2 years after the outbreak of COVID‐19 pandemic.

**Conclusions:**

Although it has been more than 2 years after the COVID‐19 pandemic outbreak, the mental health of medical staff remains a concern. In particular, medical staff involved in the clinical care of COVID‐19 patients showed a higher risk of PTSD and depression 2 years after the COVID‐19 pandemic. This study may provide some useful suggestions for psychological interventions for medical staff.

## Graphical abstract

Prevalence of PTSD/depression among medical staff 2 years after the COVID‐19 pandemic.

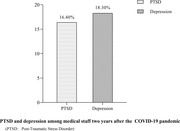

## 1 INTRODUCTION

In December 2019, coronavirus disease 2019 (COVID‐19) was first reported in Wuhan, China (Zhou et al., 2020). In order to control the spread of the COVID‐19, Wuhan was blocked for up to 76 days. This was the largest public health quarantine policy adopted in the Chinese history (Du et al., 2020). Epidemiologists believed that COVID‐19 and quarantine policy may place a high degree of psychological stress on medical staff (Baloch et al., 2021; Li et al., 2021). Medical staff in such environments are at high risk for mental health problems and disorders, particularly posttraumatic stress disorder (PTSD) and depression (Carmassi et al., 2020; Xiang et al., 2020). PTSD and depression caused a number of problems to the physical and mental health of medical staff, reducing their quality of life and even increasing the risk of suicidal behavior (Ozdemir et al., 2015). During the COVID‐19 pandemic, PTSD, depression, and other adverse mental issues may also have long‐term effects on the work performance and job satisfaction of medical staff, increasing burnout and turnover intention, leading to a decrease in the quality of medical services and COVID‐19 pandemic prevention and control (Şahin et al., 2020; Testoni et al., 2022; Zhu et al., 2022).

As the negative economic and social impact of the COVID‐19 pandemic becomes apparent, PTSD and depression will relapse or even worsen (Ransing et al., 2020). This was corroborated by a recent study that reviewed 44 articles on Severe Acute Respiratory Syndrome (SARS), Ebola, and Middle East Respiratory Syndrome (MERS) and found that psychiatric symptoms among medical staff during outbreaks may persist for a considerable period of time, with PTSD symptoms lasting 1–3 years in 10%–40% of participants (Preti et al., 2020). Timely mental health assessment at different points during the COVID‐19 pandemic is critical.

At the beginning of the COVID‐19 pandemic outbreak, many researchers explored studies of the psychological impact of COVID‐19 on medical staff (Liu et al., 2020; Song et al., 2020). However, to our knowledge, there was no studies that investigated symptoms of PTSD and depression among medical staff 2 years after the COVID‐19 pandemic outbreak in Wuhan, China. In addition, medical staff in Wuhan witnessed the high mortality and morbidity caused by COVID‐19 during the COVID‐19 pandemic, which may cause long‐lasting and severe physical and psychological trauma (Xu et al., 2022). Therefore, more studies are warranted to fully understand the impact of the COVID‐19 pandemic on PTSD and depression among medical staff in Wuhan over a longer period of time.

This study aimed to evaluate the prevalence and related factors of PTSD and depression among medical staff 2 years after the COVID‐19 outbreak in Wuhan, China, providing empirical evidence to conduct psychological intervention strategies for medical staff.

## 2 METHODS

### 2.1 Ethics statement

The study was conducted according to the guidelines of the Declaration of Helsinki, and approved by the Institutional Review Board (or Ethics Committee) of the Research Ethics Committee in Tongji Medical College, Huazhong University of Science and Technology, Wuhan, China.

### 2.2 Study design

The present multicenter study was reported as the Strengthening the Reporting of Observational Studies in Epidemiology (STROBE) guidelines for cross‐sectional studies (von Elm et al., 2007).

### 2.3 Participants

We recruited 1795 medical staff from five general hospitals in Wuhan in this survey from December 1, 2021 to December 28, 2021. In this study, the sampling process included two stages. In the first stage, five hospitals in Wuhan were randomly selected based on a simple random principle. In the second stage, online questionnaires were sent to the medical staff of the five selected hospitals through the department of medical office and the nursing department. The participant inclusion criteria were as follows: participants were (1) officially registered medical staff in China; (2) full‐time medical staff, including doctors, nurses, administrators, and so forth; and (3) informed and agreed to participate in this study. Participants with a history of PTSD, depression, or other previously diagnosed psychological disorders, participants taking medication for psychological disorders, and participants with obvious logical data errors after the examination were excluded from this study.

### 2.4 Data collection

A total of 1795 medical staff voluntarily completed the online survey, with a response rate of 97.03%. General demographic characteristics, social support, and mental health statuses such as PTSD and depressive symptoms were included in the online questionnaires. All participants completed a questionnaire on an online survey platform (“SurveyStar,” Changsha Ranxing Science and Technology, Shanghai, China). Before registration, all participants supplied electronic informed consent. Two options (yes/no) were available on the informed consent page. Only participants who picked “yes” were taken to the next page. Each verified account was eligible to answer only once to prevent repeated questionnaires. Simultaneously, intelligent logic examinations were set up in the software to identify inoperative questionnaires. Two independent researchers checked the answers to all valid questionnaires automatically entered into a data file.

### 2.5 Measurements

PTSD was assessed using the PTSD Checklist‐5 (PCL‐5) (Blevins et al., 2015), which included 20 items. The score of each item is 0–4 points, ranging from “never” to “serious.” Possible scores range from 0 to 80 points. Participants’ scores were added together, with totals positively correlated with PTSD severity—a higher score on the scale indicates higher levels of PTSD. The cutoff point for PTSD was a total PCL‐5 score of ≥33 (Ouyang et al., 2022; Song et al., 2020). The PCL‐5 scale was validated by the developers with a good internal consistency (Cronbach's alpha = .94) and test–retest (*r* = .82) (Blevins et al., 2015). The reliability and validity of the Chinese edition was confirmed (Wang et al., 2015). In our current sample, Cronbach's alpha value was .954, indicating high reliability.

Depression was surveyed using the Center for Epidemiologic Studies Depression Scale (CES‐D) (Radloff, 1991). This is a 20‐item scale with items rated on a 4‐point Likert scale (from 0 = *almost none of the time, less than one day* to 3 = *all of the time, 5−7 days*). Depression was defined if the total score of CES‐D was ≥16 (Chin et al., 2015). The CES‐D was found to has satisfactory internal consistency with a Cronbach's alpha of .8 and good retest reliability (Radloff, 1991). Additionally, the Chinese edition of CES‐D was validated and widely applied in psychological research (Zhang et al., 2015). In this study, Cronbach's alpha was .962.

The Multidimensional Scale of Perceived Social Support (MSPSS) (Zimet et al., 1988) was used to assess participants’ social support structures. The scale includes 12 items with responses ranging from 1 (*strongly disagree*) to 7 (*strongly agree*). It evaluated the quality of social support received from friends, family, and significant others. All items were aggregated and then separated by 12. Source of support means ranging from 1 to 2.99, 3 to 5, and 5.01 to 7 were categorized as low, medium, and high perceived support levels, respectively (Zimet et al., 1988). The Chinese version of the MSPSS has shown benefit reliability, and validity in disparate populations (Feng et al., 2018). The reliability and stability of the MSPSS as reported by the developers were strong, with the Cronbach's alpha and test–retest ranging from .85 to .91 and .72 to .85 for each subscale and the scale as a whole, respectively (Zimet et al., 1988). In this study, the Cronbach's alpha values for the three dimensions of the scale—family, friends, and significant others—were .960, .968, and .940, respectively.

### 2.6 Statistical analysis

Categorical data were reflected as number and percentage, while successive data were reflected as mean ± SD. The Chi‐square (*χ* ^2^) test or Fisher's exact test was used to analyze categorical data. Mann–Whitney–Wilcoxon test and Kruskal–Wallis test were implemented to compare nonnormal continuous variables. The Mann–Whitney–Wilcoxon test analyzed the difference between two groups, while the Kruskal–Wallis test was used to compare more than two groups. Multivariate adjusted stepwise logistic regression models were adopted to estimate the association between the various factors and PTSD and depression. All statistical analyses were conducted using SPSS19.0 (SPSS Inc, Chicago, IL, USA). A value of *p* < .05 (two‐tailed) was considered statistically significant.

## 3 RESULTS

A total of 1795 medical staff participated in this study, of which 1544 (86.02%) were female and 251 (13.98%) were male. A total of 1289 (71.81%) were married, and the remaining 506 (28.19%) were single. Among the participants, 295 (16.40%) and 329 (18.30%) met the criteria for PTSD and depression, respectively. It was important to note that social support was significantly associated with the risk of PTSD and depression, respectively (*p* < .05) (Table 1). The mean PTSD score was 24.18 ± 11.72. The mean value of the depression score was 10.85 ± 9.96. However, friends support was not significantly associated with depression scores (*p* < .081) (Table 2).

**TABLE 1:** Differences in the prevalence of PTSD and depression among/between various sociodemographic and other characteristic subgroups for medical staff

Variables	Total (*n* = 1795)	PTSD	*χ* ^2^	*p*	Depression	*χ* ^2^	*p*
Age (years), *n* (%)			6.774	.034		9.445	.009
18–29	571 (31.81)	75 (13.10)			97 (17.00)		
30–39	912 (50.81)	166 (18.20)			190 (20.80)		
≥40	312 (17.38)	54 (17.30)			42 (13.50)		
Sex, *n* (%)			20.658	<.001		8.937	.003
Female	1544 (86.02)	229 (77.60)			266 (17.20)		
Male	251 (13.98)	66 (14.80)			63 (25.10)		
Marital status, *n* (%)			8.143	.004		0.094	.760
Single	506 (28.19)	63 (12.50)			95 (18.80)		
Married	1289 (71.81)	232 (18.00)			234 (18.20)		
Participated in COVID‐19 clinical work, *n* (%)			5.899	.042		6.167	.038
No	868 (48.36)	132 (15.20)			147 (16.90)		
Yes	927 (51.64)	163 (17.60)			182 (19.50)		
Working environment, *n* (%)			48.835	<.001		71.22	<.001
Safe	1146 (63.84)	135 (11.80)			145 (12.70)		
General	410 (22.84)	93 (22.70)			108 (16.30)		
Unsafe	239 (13.31)	67 (28.00)			76 (31.80)		
Doctor–patient relationship, *n* (%)			64.273	<.001		79.515	<.001
Good	362 (20.17)	110 (30.40)			204 (14.20)		
Poor	1433 (79.83)	185 (12.90)			125 (34.50)		
Physical exercise, *n* (%)			7.971	.005		6.443	.011
No	1330 (74.09)	238 (17.90)			262 (19.70)		
Yes	465 (25.91)	57 (12.30)			67 (14.40)		
Health status, *n* (%)			79.513	<.001		125.141	<.001
Health	1324 (73.76)	139 (29.50)			162 (12.20)		
Unhealth	471 (26.24)	156 (11.80)			167 (35.50)		
Job satisfaction, *n* (%)			65.073	<.001		94.09	<.001
Satisfied	1499 (83.51)	201 (13.40)			218 (14.50)		
General	223 (12.42)	65 (29.10)			76 (34.10)		
Dissatisfied	73 (4.07)	29 (39.70)			35 (47.90)		
Social support							
Family support, *n* (%)			84.198	<.001		257.552	<.001
High	1269 (70.70)	144 (11.30)			117 (9.20)		
Moderate	431 (24.01)	129 (29.90)			188 (43.60)		
Low	95 (5.29)	22 (23.20)			24 (25.30)		
Friends support, *n* (%)			93.527	<.001		228.339	<.001
High	1156 (64.40)	119 (10.30)			98 (8.50)		
Moderate	553 (30.81)	159 (28.80)			214 (38.70)		
Low	86 (4.79)	17 (19.80)			17 (19.80)		
Coworkers and others support, *n* (%)			105.674	<.001		240.879	<.001
High	1188 (65.54)	121 (10.20)			99 (8.40)		
Moderate	522 (29.08)	159 (30.50)			209 (39.70)		
Low	85 (5.38)	15 (17.70)			21 (24.70)		
Total	1795 (100.00)	295 (16.40)			329 (18.30)		

Abbreviation: PTSD, posttraumatic stress disorder.

**TABLE 2:** Differences in the score of PTSD and depression among/between various sociodemographic and other characteristic subgroups for medical staff

		PTSD				Depression			
Variables	Total (*n* = 1795)	*M*	*SD*	Z/K–W	*p*	*M*	*SD*	Z/K–W	*p*
Age				34.267^b^	<.001			13.638^b^	.001
18–29	571	22.48	11.57			10.37	9.87		
30–39	912	25.11	11.92			11.60	10.29		
≥40	312	24.57	11.09			9.52	8.94		
Sex				−2.098^a^	.036			−1.026^a^	.305
Female	1544	23.82	11.34			10.68	9.74		
Male	251	26.38	13.69			11.88	1.20		
Marital status				−4.119^a^	<.001			−0.22^a^	.826
Single	506	22.64	10.99			10.93	9.99		
Married	1289	24.79	11.95			10.82	9.95		
Participated in COVID‐19 clinical work				−0.852^a^	.394			−2.462^a^	.041
No	868	23.82	11.30			10.52	9.82		
Yes	927	24.52	12.10			11.14	10.08		
Working environment				113.169^b^	<.001			93.305^b^	<.001
Safe	1146	22.06	9.87			9.06	8.27		
General	410	27.06	13.60			13.15	11.21		
Unsafe	239	29.42	13.64			15.44	12.52		
Doctor–patient relationship				−9.618^a^	<.001			−8.99^a^	<.001
Good	362	22.74	10.47			9.65	8.88		
Poor	1433	29.88	14.41			15.57	12.34		
Physical exercise				−3.608^a^	<.001			−4.136^a^	<.001
No	1330	24.60	11.65			11.24	9.99		
Yes	465	22.97	11.87			9.72	9.77		
Health status				−11.796^a^	<.001			−13.413^a^	<.001
Health	1324	22.30	10.39			8.97	8.43		
Unhealth	471	29.46	13.51			15.13	11.86		
Job satisfaction				70.133^b^	.527			76.149^b^	<.001
Satisfied	1499	23.04	10.79			9.77	8.86		
General	223	29.55	14.11			15.13	12.41		
Dissatisfied	73	23.04	10.79			19.85	14.12		
Social support									
Family support				5.654^b^	.017			4.008^b^	.045
High	1269	22.02	9.93			12.54	12.83		
Moderate	431	30.11	13.69			19.85	14.12		
Low	95	26.13	14.43			17.76	11.54		
Friends support				6.375^b^	.041			5.021^b^	.081
High	1156	21.59	9.79			8.03	7.72		
Moderate	553	29.41	13.35			16.65	11.28		
Low	86	25.36	13.10			11.33	11.28		
Coworkers and others support				176.976^b^	<.001			267.504^b^	<.001
High	1188	21.62	9.70			8.10	7.58		
Moderate	522	29.73	13.41			16.87	11.58		
Low	85	25.40	14.10			11.69	11.63		
Total	1795	24.18	11.72			10.85	9.96		

Abbreviation: PTSD, posttraumatic stress disorder.

^a^ Z: Mann–Whitney *U*‐test

^b^ K–W: Kruskal–Wallis test.

In the multivariate adjusted logistic regression analysis (Table 3), the following factors were independently associated with PTSD among medical staff 2 years after the COVID‐19 outbreak: male (adjusted odds ratio [OR] = 1.92, 95% confidence interval [CI]: 1.37–2.7, *p* ≤ .001), married (adjusted OR = 1.66, 95% CI: 1.20–2.28, *p* < .001), involved in the COVID‐19 clinical work (adjusted OR = 2.09, 95% CI: 1.22–3.63, *p* = .009), unsafe working environment (adjusted OR = 1.25, 95% CI: 1.04–1.51, *p* = .021), poor doctor–patient relationship (adjusted OR = 1.60, 95% CI: 1.16–2.21, *p* = .004), being unhealthy (adjusted OR = 2.23, 95% CI: 1.68–2.96, *p* < .001), work dissatisfaction (adjusted OR = 1.43, 95% CI: 1.13–1.82, *p* = .003), and low levels of family support (adjusted OR = 1.72, 95% CI: 1.39–2.13, *p* ≤ .001).

**TABLE 3:** Multivariate adjusted logistic regression of PTSD and depression

Variables	*β*	*SE*	Wald	Adjusted OR	95% CI	*p*
PTSD						
Constant	−6.546	0.477	188.411			<.001
Sex	0.654	0.173	14.292	1.92	1.37–2.70	<.001
Married	0.504	0.173	14.292	1.66	1.20–2.28	<.001
Participated in COVID‐19 clinical work	0.739	0.281	6.901	2.09	1.22–3.63	.009
Unsafe working environment	0.221	0.096	5.362	1.25	1.04–1.51	.021
Poor doctor–patient relationship	0.472	0.163	8.344	1.60	1.16–2.21	.004
Unhealth	0.802	0.144	31.071	2.23	1.68–2.96	<.001
Work dissatisfaction	0.359	0.122	8.581	1.43	1.13–1.82	.003
Low family support	0.540	0.109	24.403	1.72	1.39–2.13	<.001
Depression						
Constant	−6.355	0.391	264.511			<.001
Participated in COVID‐19 clinical work	0.289	0.136	4.5	1.33	1.02–1.74	.034
Unsafe working environment	0.231	0.095	5.888	1.26	1.05–1.52	.015
Poor doctor–patient relationship	0.408	0.162	6.323	1.50	1.09–2.07	.012
Unhealth	0.989	0.142	48.644	2.69	2.04–3.55	<.001
Work dissatisfaction	0.418	0.122	11.675	1.52	1.20–1.93	.001
Low family support	0.463	0.161	8.282	1.59	1.16–2.18	.004
Low coworkers and others support	0.657	0.165	15.806	1.93	1.40–2.67	<.001

Abbreviations: CI, confidence interval; PTSD, posttraumatic stress disorder.

Table 3 shows the association between various factors and the risk of depression among medical staff. Participation in COVID‐19 clinical work (adjusted OR = 1.33, 95% CI: 1.02–1.74, *p* = .034), unsafe working environment (adjusted OR = 1.26, 95% CI: 1.05–1.52, *p* = .015), poor doctor–patient relationship (adjusted OR = 1.50, 95% CI: 1.09–2.07, *p* = .012), being unhealthy (adjusted OR = 2.69, 95% CI: 2.04–3.55, *p* < .001), work dissatisfaction (adjusted OR = 1.52, 95% CI: 1.20–1.93, *p* = .001), low levels of family support (adjusted OR = 1.59, 95% CI: 1.16–2.18, *p* = .004), and low co‐workers and others support (adjusted OR = 1.93, 95% CI: 1.40–2.67, *p* < .001) were independently associated with depression 2 years after the COVID‐19 pandemic outbreak.

## 4 DISCUSSION

To our knowledge, this was the first multicenter study that investigated the prevalence of PTSD and depression among medical staff in five general hospitals 2 years after the outbreak of the COVID‐19 pandemic in Wuhan, China. Notably, participating in COVID‐19 clinical work, unsafe work environment, female, work dissatisfaction, poor doctor–patient relationship, being unhealthy, and low levels of family support were significantly associated with the risk of PTSD and depression. These findings may provide a basis for designing future interventions for medical staff.

Compared to previous studies (Wang et al., 2021; Zhang et al., 2022), the lower rates of PTSD and depression among medical staff were reported in this study. In addition to differences of participants, this may be largely related to the period of investigation. With the implementation of effective medical and public health measures, the stress of medical staff was significantly reduced (Pan et al., 2020). However, the mental health of medical staff 2 years after the pandemic still deserves our attention.

Many susceptibility factors influence the risk and degree of PTSD and depression. However, trauma appears to be the most significant factors (Rofman, 2009). Consistent with previous study, direct involvement in COVID‐19 clinical work may be an important indicator for assessing trauma exposure (Luo et al., 2021). During the COVID‐19 pandemic, a large percentage of medical staff directly involved in COVID‐19 treatment and care reported symptoms of PTSD and depression (Luo et al., 2021; Mosheva et al., 2021). Although there was no statistical difference in PTSD scores between medical staff who did and did not directly participate in COVID‐19 clinical work, involved in COVID‐19 clinical work was significantly associated with the risk of PTSD among medical staff in this study. In addition, the prevalence of depression was high among medical staff involved in COVID‐19 clinical work, similar to that found in previous studies (Song et al., 2020; Wang et al., 2021). Similarly, participants in unsafe working environments had a higher risk of PTSD and depression. There was the additional hazard of spreading infectious illness interdepartmentally when in contact with a high concentration of patients with COVID‐19. Medical staff more regularly came into contact with confirmed COVID‐19 cases. This contact often occurred while providing basic nursing services to patients, especially in the respiratory, emergency, and intensive care departments. This may be attributed to the high risk of COVID‐19 infections and the fear among medical staff (Steudte‐Schmiedgen et al., 2021) of becoming infected with COVID‐19. Thus, those involved in frontline COVID‐19 clinical work remain a priority population for follow‐up psychological interventions.

In addition to trauma exposure with COVID‐19, demographic and other occupation‐related factors are influential in PTSD and depression in medical staff. This study showed that female respondents had a higher risk of PTSD than male respondents, consistent with the previous study conducted in the early stages of the COVID‐19 pandemic (Rodriguez et al., 2021). Additionally, women were at higher risk of PTSD than men to suffer from PTSD in their lifetime after exposure to similar traumatic occurrences (Fullerton et al., 2001). Still, these results need to be tempered with caution. Far more females participated in the survey than males, and most medical staff doing COVID‐19‐related tasks were women, which may result in a selection bias. In this study, medical staff dissatisfied with work and poor doctor–patient relationships showed a higher risk of PTSD and depression. This was consistent with Zhou et al. (2018), who implied that mental health correlates with job satisfaction. In addition, poor doctor–patient relationships can sometimes lead to workplace violence, severely affecting the mood of medical workers. Studies have shown that improving the doctor–patient relationship is critical to mitigating depression symptoms and improving patient prognosis (Gong et al., 2014; Zhou et al., 2021).

Health status and social support, particularly from family, are contributing factors that might help people adapt to challenging occupational environments (Kerai et al., 2017). In this study, medical staff who were unhealthy and had low levels of family support were more susceptible to PTSD and depression. Previous studies have also shown a strong association between physical health status and quality of life and psychological well‐being (Kao et al., 2021; Öztürk Çopur & Karasu, 2021). In accordance with the previous study, we also found that low family support was significantly associated with risk and higher scores for PTSD and depression compared to high family support during the COVID‐19 pandemic (Liu et al., 2020). In addition to family support, support from co‐workers and others in this study was a protective factor for PTSD in medical staff. A recent study also demonstrated that colleague and organizational social support can buffer the development of mental health disorders in medical staff (Riedel et al., 2021). Therefore, providing support from family and organization during the COVID‐19 pandemic may be an effective way to improve the mental health of medical staff.

The current research has some limitations. First, the participants of this study were medical staff in Wuhan, China. Wuhan was the earliest city and one of the severe cities during the COVID‐19 pandemic. This may limit generalizability to medical staff in areas with less severe COVID‐19 pandemic outbreak. Second, given the limitations of the cross‐sectional design, robust conclusions concerning the causal effect of COVID‐19 on mental health among medical staff cannot be drawn. The long‐term mental health effects of COVID‐19 on medical staff still need to be validated in additional prospective cohort studies with large samples. Finally, the proportion of female nurses is relatively large; therefore, future studies should focus on the proportion of medical staff. Despite these limitations, this multicenter study analyzed the prevalence and risk factors of PTSD and depression among medical staff 2 years after the COVID‐19 outbreak in Wuhan, China. The results could help theoretically explain PTSD and depression in this community during the COVID‐19 pandemic. Furthermore, it can provide a foundation for creating coping programs for these practitioners in future public health emergency events.

Understanding the prevalence of PTSD and depression among medical staff during the COVID‐19 pandemic is necessary to maintain a stable and productive hospital team. This empirical investigation is useful in helping hospital leaders identify at‐risk populations when conducting psychological interventions with medical staff. Medical staff directly involved in clinical work with COVID‐19 or who reported unhealthy status should be the focus of the psychiatric interventions. Besides, improving the work environment and increasing organizational/coworker care seems to be an effective way to address the PTSD and depression of medical staff.

## 5 CONCLUSIONS

During the COVID‐19 pandemic, the mental health status of medical staff is a concern. A proportion of healthcare workers still were suffering from PTSD and depression after 2 years of the COVID‐19 pandemic. Therefore, continuous psychiatric intervention is essential and urgent during the COVID‐19 pandemic. Hospital administrators must understand that they must adopt long‐term monitoring or psychiatric interventions for the long‐term well‐being of medical staff, especially those who directly involved in COVID‐19 clinical work or reported unhealthy conditions.

## AUTHOR CONTRIBUTIONS

Wenning Fu and Yifang Liu conceptualized the idea of the study and wrote the original draft of the manuscript. Li Zou designed methodology. Xue Bai provided software and administered the project. Pu Zhang and Jun Zhang performed validation and curated the data. Li Zou and Jing Wen performed formal analysis. Wenning Fu, Yifang Liu, Li Zou, and Jing Wen performed investigation. Jing Wang and Jing Mao provided resources, reviewed and edited the manuscript, performed supervision, and acquired funding. All authors have read and agreed to the published version of the manuscript.

## CONFLICT OF INTEREST

The authors declare no conflict of interest.

### PEER REVIEW

The peer review history for this article is available at https://publons.com/publon/10.1002/brb3.2785

## Data Availability

The data presented in this study are available on request from the corresponding author.
